# Regional lymphadenectomy vs. extended lymphadenectomy for hilar cholangiocarcinoma (Relay-HC trial): study protocol for a prospective, multicenter, randomized controlled trial

**DOI:** 10.1186/s13063-019-3605-z

**Published:** 2019-08-23

**Authors:** Min He, Xinsen Xu, Hao Feng, Wei Chen, Houbao Liu, Yongjie Zhang, Jianming Wang, Zhimin Geng, Yudong Qiu, Weidong Duan, Xiangcheng Li, Xuting Zhi, Weihua Zhu, Fuyu Li, Jiangtao Li, Shengping Li, Yu He, Zhiwei Quan, Jian Wang

**Affiliations:** 1 Department of Biliary-Pancreatic Surgery, Renji Hospital affiliated to Shanghai Jiao Tong University School of Medicine, Pujian Road 160, Shanghai, 200127 People’s Republic of China; 2 Department of General Surgery, Ruijin Hospital, School of Medicine, Shanghai Jiao Tong University, Shanghai, 200025 People’s Republic of China; 3 Department of General Surgery, Zhongshan Hospital, School of Medicine, Shanghai Fu Dan University, Shanghai, 200032 People’s Republic of China; 4 Department of Biliary Surgery, The Third Affiliated Hospital, The Second Military Medical University, Shanghai, 200438 People’s Republic of China; 5 Department of General Surgery, Tongji Medical College, Hua Zhong University of Science&Technology, Hubei, 430030 People’s Republic of China; 6 Department of General Surger, The First Affiliate Hospital of Xi An Jiao Tong University, Shaanxi, 710061 People’s Republic of China; 7 Department of General Surgery, Nanjing Drum Tower Hospital, The Affiliated Hospital of Nanjing University Medical School, Jiangsu, 210008 People’s Republic of China; 8 Department of General Surgery, Chinese PLA General Hospital, Medical School of Chinese PLA, Beijing, 100853 People’s Republic of China; 9 Department of General Surgery, The First Affiliated Hospital with Nan Jing Medical University, Jiangsu, 210029 People’s Republic of China; 10 Department of General Surgery, Qilu Hospital of Shandong University, Shandong, 250012 People’s Republic of China; 11 Department of General Surgery, Peking University People’s Hospital, Beijing, 100044 People’s Republic of China; 12 Department of General Surgery, West China Hospital Sichuan University, Sichuan, 610041 People’s Republic of China; 13 Department of General Surgery, The Second Affiliated Hospital of Zhejiang University School of Medicine, Zhejiang, 310009 People’s Republic of China; 14 Department of General Surgery, Sun Yat-Sen University Cancer Center, Guangdong, 510060 People’s Republic of China; 15 Department of General Surgery, The First Hospital Affiliated to AMU (Southwest Hospital), Chongqing, 400038 People’s Republic of China; 16 Department of General Surgery, Xinhua Hospital affiliated to Shanghai Jiao Tong University School of Medicine, Kongjiang Road1665, Shanghai, 200092 People’s Republic of China

**Keywords:** Hilar cholangiocarcinoma, Regional lymphadenectomy, Extended lymphadenectomy

## Abstract

**Background:**

The prognostic benefits and safety of extended lymphadenectomy for hilar cholangiocarcinoma remain uncertain. The available evidence is still insufficient concerning its retrospective aspect. The aim of this study is to explore the clinical effect and safety of extended lymphadenectomy compared to regional lymphadenectomy in patients with hilar cholangiocarcinoma.

**Methods:**

The Relay-HC trial is a prospective, multicenter, and randomized controlled trial. Seven hundred and thirty-four eligible patients with resectable perihilar cholangiocarcinoma across 15 tertiary hospitals in China will be randomly assigned (1:1) to receive either regional lymphadenectomy or extended lymphadenectomy. The primary objective is to determine the overall survival after the two approaches. Secondary objectives of the study include the evaluation of perioperative mortality, postoperative complication, and disease-free survival. This study has been approved by the ethics committee of each participating hospital.

**Discussion:**

The Relay-HC trial is designed to investigate the prognostic benefits and safety of expanded lymphadenectomy for hilar cholangiocarcinoma. Currently, it has never been investigated in a prospective randomized controlled clinical trial.

**Trial registration:**

Chinese Clinical Trial Registry (ChiCTR), ChiCTR1800015688. Registered on 15 April 2018.

**Electronic supplementary material:**

The online version of this article (10.1186/s13063-019-3605-z) contains supplementary material, which is available to authorized users.

## Background

Hilar cholangiocarcinoma is one of the most common bile duct cancers, accounting for 60–70% of extrahepatic cholangiocarcinomas. Surgical resection remains the mainstay of potentially curative treatment for hilar cholangiocarcinoma. However, the probability of radical curative resection is low, and the prognosis is insufficient [1–3].

The incidence of lymph node metastases is high on the presentation or exploratory laparotomy. As the prognosis of patients with nodal metastases is significantly worse, the American Joint Committee on Cancer (AJCC) has modified the staging of lymph nodes invasion (N) several times during the last decade. However, concerning lymphatic dissection, there are still many disputes [1, 4, 5], and currently, no consensus has been reached on the preferred method of lymphadenectomy in patients with hilar cholangiocarcinoma. The incidence of complications after extended and regional lymphadenectomy has only been assessed in small retrospective series.

We hypothesize that extended lymph node dissection (8a/p, 9, 12a/b/c/h/p, 13a, 14, 16) might improve the prognosis of the patients without elevating the major complication rate. Therefore, the primary objective is to determine the overall survival (OS) rate for the two approaches with the secondary endpoint of perioperative mortality, postoperative complications, and disease-free survival (DFS). This study will explore the prognostic benefits and safety of extended lymphadenectomy for hilar cholangiocarcinoma.

## Methods

### Design

The Relay-HC trial is a prospective, multicenter, randomized controlled trial. Patients who will receive curative radical resection for hilar cholangiocarcinoma would be randomly assigned (1:1) to receive either regional lymphadenectomy or extended lymphadenectomy. The sample size calculation resulted in a requirement of 734 patients. On the basis of previous data, the median 5-year OS of patients with hilar cholangiocarcinoma who underwent regional lymphadenectomy (P1) was 0.17 (0.07–0.20) [6–9], and the 5-year OS for those with extended lymphadenectomy (P2) was 0.26 [3, 10, 11]. The α level (type I error) is set as 0.05 (one-sided), β is set as 0.2, and the power is set as 0.8. The formula to calculate the sample quantity for a high-quality clinical trial is [12]:

$$ {n}_1={n}_2=\frac{\Big[{u}_a\sqrt{2p\left(1-p\right)}+{u}_{\beta}\sqrt{p_1\left(1-{p}_1\right)+{p}_2\left(1-{p}_2\right)\Big]{}^2}}{{\left({p}_1-{p}_2\right)}^2} $$

The minimum sample size for each group is 330. As the actual sample size includes 10% shedding, the actual sample size of each group is thus 367 patients.

### Case selection

All patients aged between 18 and 80 years old with hilar cholangiocarcinoma would be referred for a multidisciplinary team evaluation at each center. Hilar cholangiocarcinoma is defined as a cholangiocarcinoma that develops at the point where the left and right hepatic ducts join to form the common hepatic duct (as determined by computed tomography [CT] imaging or magnetic resonance cholangiopancreatography, or both). Criteria for resectable hilar cholangiocarcinoma include an anticipated complete (R0) resection with adequate future liver remnant (FLR > 30%) and Child-Turcotte-Pugh grade A–B, as well as American Society of Anesthesiologists (ASA) grades I–III. The residual liver volume will be assessed by three-dimensional reconstruction of CT images. These procedures will be accomplished by professional radiographers and the multidisciplinary teams from each center, who will be trained at the leading affiliation to ensure standardization. Patients who also have other malignancies would be excluded from the study.

### Setting

**Fig. 1:**
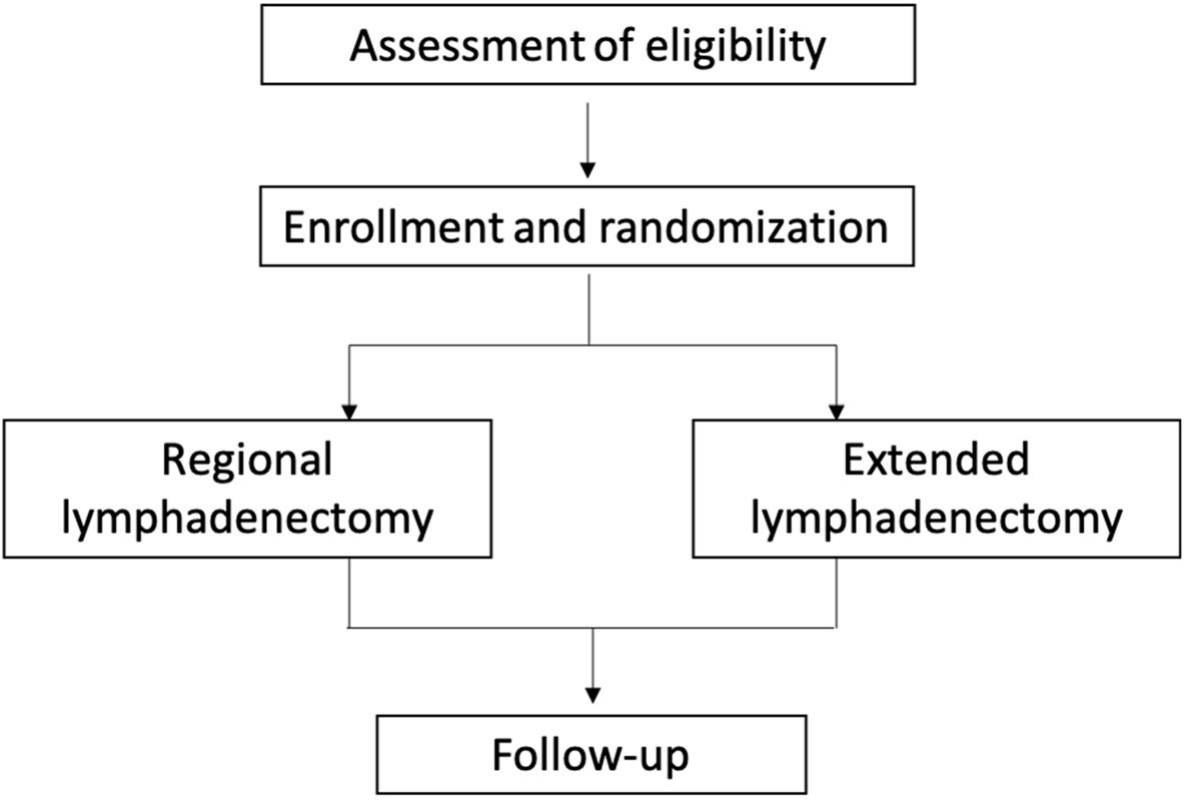
A flowchart of the study design

The study is performed at hepatobiliary surgery centers from 15 tertiary hospitals that receive the majority of patients with hilar cholangiocarcinoma from various parts of China. Each of the centers is qualified for standard radical resection of hilar cholangiocarcinoma and regional/extended lymph node dissection. The institutional review board at each hospital has approved the protocol. The number of cases for each center is allocated according to the number of annual cholangiocarcinoma surgeries. A flowchart of the study design was shown in Fig. 1.

A Consolidated Standards of Reporting Trials (CONSORT) checklist for this study is provided in Additional file 2. The Standard Protocol Items: Recommendations for Interventional Trials (SPIRIT) checklist is provided in Additional file 3.

### Randomization

Patients who will receive curative radical resection for hilar cholangiocarcinoma would be randomly assigned (1:1) to regional lymphadenectomy or extended lymphadenectomy by computer-generated allocation based on the envelope method and the hierarchical block randomization method. The envelopes are sealed, opaque, and sequentially numbered. Randomization is performed by the trial coordinator (Study Group of Biliary Surgery of the Surgery Branch of the Chinese Medical Association). The random number table and the block assignment number table will be kept confidential by the full-time secretary of this project. Center-stratified block-permuted randomization is used in this trial. Trial participants are subdivided into strata such as centers; then permuted block randomization is used for each stratum with a block size of 4.

### Procedure

The routine approach for hilar cholangiocarcinoma surgery is based on the National Comprehensive Cancer Network (NCCN) Guidelines Insights: Version 2.2019 Hepatobiliary Cancers [13]. The surgical procedures consist of (extended) hemi-hepatectomy and complete lymphadenectomy of the hepatoduodenal ligament. The (extended) hemi-hepatectomy contains en bloc excision of the liver hilum, extrahepatic bile ducts, and caudate lobe. Portal vein excision and reconstruction would be performed when the tumor infiltrates into the portal vein bifurcation. In this study, we will utilize the lymph node classification system by the Liver Cancer Study Group of Japan (Additional file 1: Figure S1) [14]. Patients in the regional lymph node dissection group will receive radical resection and numbers 8a/p, 12a/b/c/h/p, 13a lymph nodes dissection. In contrast, patients in the extended lymph node dissection group will receive radical resection and numbers 8a/p, 9, 12a/b/c/h/p, 13a, 14, 16 lymph nodes dissection. Experienced surgeons in each center will be educated in a standardized surgical approach, especially the surgical procedures and lymph node harvest procedures. There will be digital recording (video and photography) for each operation, which will be evaluated by third-party surgical experts, namely the data monitoring committee (DMC). The intraoperative evaluation, safety assessment, and tumor specimens will be evaluated by the surgical teams who perform the surgery as well as pathologists.

### Blinding

Concerning random allocation, a sealed envelope will be issued to the surgeon before the operation by a project secretary. Normally there are two secretaries who are recruited for the clinical trial data management who are not involved in the operation. Then the surgeon unpacks the envelope and performs the operation according to the allocation in the envelope. The patients, outcome assessors, pathologists, and data analysts will not know the grouping information.

### Follow-up

To improve data quality, double data entry would be performed by the two secretaries. Final study follow-up is scheduled at 30 days after surgery, including perioperative mortality and operative complication evaluation. Long-term follow-up assessments including tumor markers, chest radiographs, upper abdomen enhanced CT, and survival status are scheduled at 6 months, 1 year, 3 years, and 5 years after surgery.

### Observation indices

**Fig. 2:**
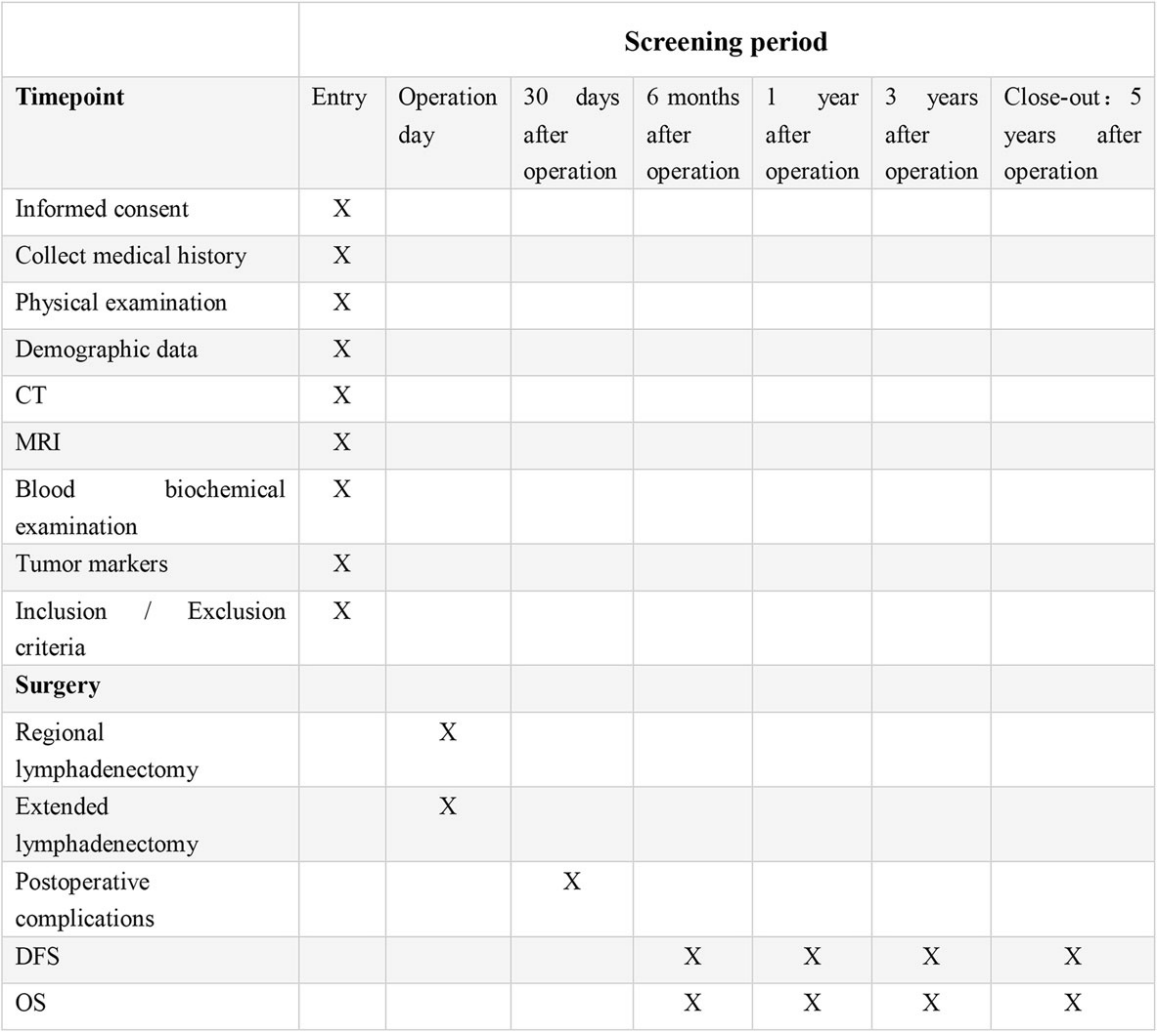
Enrollment, intervention, and assessments in the Relay-HC trial

The primary endpoint will be the 5-year OS rate. The secondary efficacy index includes the primary complication (Clavien-Dindo grades > II) within 30 days postoperatively, perioperative mortality, 6-month OS and DFS, 1-year OS and DFS, 3-year OS and DFS, and 5-year DFS. Cancer-specific survival is determined at the time of cholangiocarcinoma-related death. Disease-free survival is the time to any recurrence. The efficacy evaluation is based on the postoperative pathology: if the postoperative pathological margin is negative and LN ≥ 6, the operation is considered to have achieved the desired purpose. The safety index includes vital signs, adverse events, and clinical laboratory parameters (blood routine, urine routine, myocardial enzymes, coagulation, blood biochemistry, electrocardiogram, cardiac ultrasound), as well as early withdrawal (Fig. 2).

Specifically, the intraoperative evaluation includes the length of the operation, intraoperative hemorrhage, intraoperative blood transfusion, time of hepatic occlusion, vascular anastomosis, the area and number of lymph nodes dissected, and so on.

Concerning the tumor-related evaluation, tumor specimens will be sent for pathological evaluation about the quality of the specimens, grading, pathological stage, tumor embolus in vein, perineuronal invasion, lymph node collection, and the ratio of positive lymph nodes.

Concerning feasibility and safety, routine blood examination, hepatic and renal function, and the biochemical test will be performed the day after the surgery and then every 3 days. Chest and upper and lower abdomen CT will be performed on the seventh postoperative day to evaluate pleural effusion, lung infection, ascites, and abdominal infection. Additionally, vital signs and drainage will be monitored.

### Statistical analysis

Concerning the primary endpoint (overall survival), the log-rank test for univariable testing and Cox regression would be used to compare the long-term prognosis among patients in the extended lymphadenectomy group and the regional lymphadenectomy group in the intention-to-treat population. Secondary endpoints include DFS, survival without recurrence of regional nodal metastases, distant metastasis-free survival, the primary complication within 30 days after surgery (Clavien-Dindo grade > II), and perioperative mortality. Time zero is set as the time of randomization. After enrollment, all patients who have been randomized would be included in the full analysis set (FAS). On the basis of the FAS, patients who meet the inclusion and exclusion criteria (Table 1), are compliant, and do not violate the clinical trial protocol would form the per protocol set (PPS).

**Table 1:** Eligibility criteria in Relay-HC trial

Inclusion criteria	
1. Age > 18 years old and ≤ 80 years old	
2. Diagnosis of hilar cholangiocarcinoma by preoperative imaging and laboratory examination; confirmed as biliary malignant tumor by intraoperative frozen and postoperative pathology; evaluated as resectable by preoperative imaging	
3. Preoperative CT/MRI shows no obvious lymph node metastasis of groups 9, 14 and 16	
4. Child-Turcotte-Pugh A–B grade of liver function	
5. Residual liver volume > 30% 6. ASA grade 1–3	
7. The patient has self-care ability, understands and voluntarily signs the written informed consent, and is able to complete the follow-up plan	
8. Female patients of gestational age must be excluded from pregnancy	
9. The patient has signed the written informed consent form before the screening test	
Exclusion criteria	
1. Tumor R1 resection	
2. The patient has obvious heart, lung, brain, or kidney dysfunction that affects the treatment of cholangiocarcinoma	
3. The patient has a history of other malignancies	
4. Child-Turcotte-Pugh C grade of liver function	
5. ASA grade 4–6	
6. Females who are pregnant or breastfeeding	
7. Patients who are not suitable for the study as determined by the researcher	

The principal analysis consists of an intention-to-treat comparison of the major complications in both groups, using a Mann-Whitney *U* test for ordered categorical data with a two-sided 0.05 significance level. The proportion of patients with any severe operation-related complication will also be expressed in terms of relative risk (RR) and 95% confidence interval (CI). Categorical variables are evaluated using Pearson’s χ^2^ test or Fisher’s exact test.

## Discussion

The main factors that affect the prognosis include cellular differentiation, perineural infiltration, and lymphatic and microvascular infiltration. Lymph node metastasis is an important factor leading to poor prognosis. It is reported that the rate of lymph node metastasis in hilar cholangiocarcinoma is 20–50%. When cholangiocarcinoma has already spread to the lymph node, the 5-year survival rate declines to 5% [1–3]. At present, lymphatic dissection for hilar cholangiocarcinoma remains controversial. In the seventh edition of the AJCC hilar cholangiocarcinoma guidelines, “N” staging was based on the lymph node infiltrating range, suggesting that the area of lymph node metastasis is an important parameter for hilar cholangiocarcinoma prognosis [4]. Several other studies confirmed that the survival rate of hilar cholangiocarcinoma patients with para-aortic lymph node metastasis (N2 in the seventh phase of AJCC) was significantly shorter than that for patients with regional metastasis (N1 in the seventh phase of the AJCC). The 5-year survival rate was only 0–12% [1]. In contrast, other researchers found that patients with regional or para-aortic lymph node metastasis have similar survival rates. This contradiction provokes more clinical research to explore the correlation between lymph node status and long-term outcome. The eighth edition of the AJCC staging system redefines the “N” staging based on the number of lymph node metastases. It reflects the prognostic value of the positive lymph nodes number and leads to higher demands for the dissection range of lymph nodes. Further studies are needed to investigate the sufficient number of lymph nodes’ dissection in order to harvest an accurate number of positive lymph nodes.

Whether the extended lymph node dissection could improve the prognosis of patients with hilar cholangiocarcinoma is yet unknown. The elevated comorbidity rate induced by extended dissection has always been a major concern for hepatobiliary surgeons. Hakeem et al. found that the 3-year and 5-year OS for regional lymphadenectomy were 41% and 31%, and the 3-year and 5-year OS for extended lymphadenectomy were 26% and 12% [10]. There was no significant difference in median OS and DFS between the two groups, suggesting no prognosis benefit in extended lymphadenectomy. Kitagawa et al. showed that the OS of patients who received regional lymphadenectomy plus para-aortic lymph node dissection was significantly better than that for those who only received regional lymphadenectomy [1]. The number of positive lymph nodes exceeding 20% was independent prognostic factor [15]. However, the complication rate for the former group was 63%, which was slightly higher than the rate for the regional lymphadenectomy group [16–18]. The in-depth analysis showed that the most common complications were pleural fluid and mild wound infections (Clavien-Dindo grades I–II) rather than complications such as lymphatic leakage, hemorrhage, and liver failure [19, 20]. In the preliminary study in our center, the perioperative mortality did not increase as other literature reported. Thus, the extended lymphadenectomy for hilar cholangiocarcinoma patients might be safe and feasible for a qualified hepatobiliary surgery center.

### Trial status

The protocol version number is RELAY-HC Ver5.0, which was registered on 15 April 2018 (ChiCTR1800015688). Recruitment began on 30 July 2018, and the approximate date when recruitment will be completed is 30 July 2023.

## Additional files

Additional file 1: Figure S1 Lymph node classification system used in the Relay-HC trial (JPG 437 kb)

Additional file 2: CONSORT 2010 checklist of information to include when reporting a randomized trial. (DOC 217 kb)

Additional file 3: SPIRIT 2013 checklist: recommended items to address in a clinical trial protocol and related documents. (DOC 126 kb)

## Data Availability

Not applicable.

## Abbreviations

DFS: Disease-free survival
OS: Overall survival
