# Supplementary figures and images for: Regional lymphadenectomy vs. extended lymphadenectomy for hilar cholangiocarcinoma (Relay-HC trial): study protocol for a prospective, multicenter, randomized controlled trial
Source: Trials. 2019 Aug 23;20:528. doi: 10.1186/s13063-019-3605-z (PMC6708245; doi:10.1186/s13063-019-3605-z)

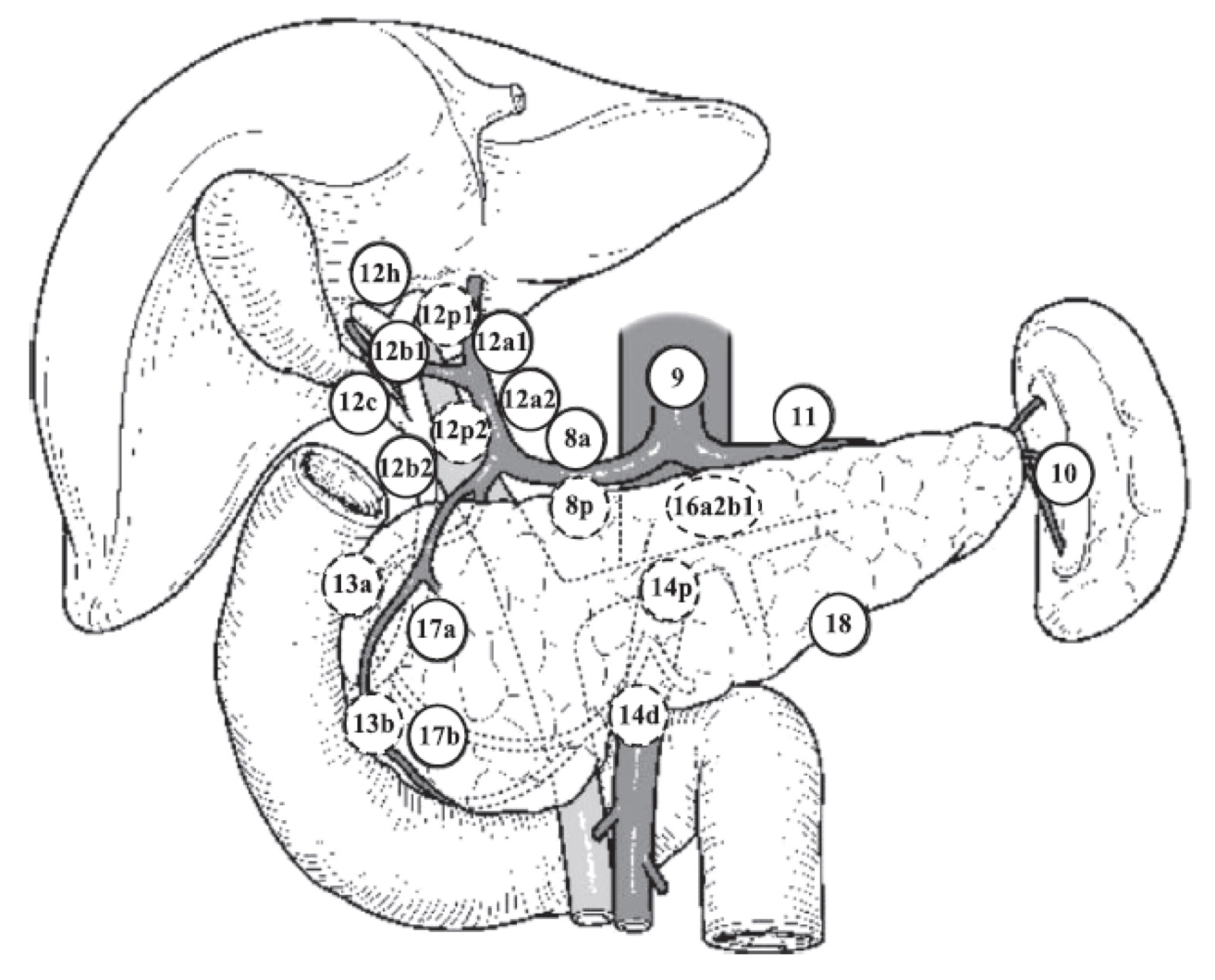

Supplement: Supplementary file 1 — Figure S1 Lymph node classification system used in the Relay-HC trial (JPG 437 kb) [file 13063_2019_3605_MOESM1_ESM.jpg]
